# Clinical characteristics and prognostic impact of Burkholderia cepacia complex colonization and infection in patients with hematologic malignancies

**DOI:** 10.3389/fcimb.2026.1728965

**Published:** 2026-01-30

**Authors:** Ziyi Liu, Yan Xie, Peiqi Liang, Dong Wang, Jie Xu, Jianhong Fu

**Affiliations:** 1 Key Laboratory of Thrombosis and Hemostasis of Ministry of Health, Department of Hematology, The First Affiliated Hospital of Soochow University, National Clinical Research Center for Hematologic Diseases (Suzhou), Jiangsu Institute of Hematology, Suzhou, China; 2 Center of Clinical Laboratory, The First Affiliated Hospital of Soochow University, Suzhou, Jiangsu, China

**Keywords:** Burkholderia cepacia complex, clinical features, colonization, hematological malignancies, infection, prognosis

## Abstract

**Objective:**

In recent years, there has been an increasing number of reports on infections caused by the Burkholderia cepacia complex (Bcc). However, the clinical characteristics and prognosis of Bcc infections in patients with hematological malignancies remain unreported. This study aimed to delineate the clinical trajectory from colonization to active infection and to identify prognostic factors influencing outcomes in hematologic malignancy patients with Bcc colonization.

**Methods:**

We conducted a retrospective analysis of 185 hematological malignancy patients with Bcc colonization at the Department of Hematology, The First Affiliated Hospital of Soochow University, from November 2019 to November 2022. This study aimed to identify independent risk factors for mortality in this patient population.

**Results:**

Respiratory failure (*P* < 0.001), hepatic insufficiency (*P* < 0.001), renal insufficiency (*P* = 0.001), and post-colonization infections (*P* = 0.01) were significantly associated with adverse prognosis in Bcc-colonized hematological malignancy patients. Post-colonization infections emerged as an independent risk factor for poor prognosis (HR = 2.855, 95%CI 1.214–6.715, *P* = 0.016). Subgroup analysis revealed that hematopoietic stem cell transplantation (HSCT) recipients with post-colonization infections had particularly poor outcomes (HR = 3.733, 95%CI 1.179–11.824, *P* = 0.025).

**Conclusion:**

Progression from Bcc colonization to active infection, particularly in HSCT recipients, signifies a critical juncture with markedly increased mortality. Implementing vigilant surveillance protocols to prevent this transition is paramount for improving survival in this vulnerable population.

## 1 Introduction

The Burkholderia cepacia complex (Bcc), a group of metabolically versatile Gram-negative bacteria, is recognized as an opportunistic pathogen notorious for its intrinsic multidrug resistance (Gautam et al., 2011). While historically linked to chronic respiratory infections in patients with cystic fibrosis (Kenna et al., 2017; Mahenthiralingam and Vandamme, 2005), Bcc has emerged as a growing cause of nosocomial outbreaks (Angrup et al., 2022; Shen et al., 2023; Sinha et al., 2023; Viderman et al., 2020), posing a significant threat to immunocompromised individuals (Ho et al., 2021; Paes Leme et al., 2022; Siddiqui et al., 2022). Patients with hematologic malignancies represent a uniquely vulnerable cohort due to profound therapy-induced immunosuppression, prolonged neutropenia, and frequent breaches of mucocutaneous barriers via indwelling catheters. Despite this heightened susceptibility, the clinical course and prognostic determinants specifically associated with Bcc colonization and subsequent infection in this population remain inadequately characterized. This study aimed to bridge this knowledge gap by analyzing one of the largest single-center cohorts to date, with a focus on identifying risk factors for mortality and the critical transition from colonization to active infection.

## 2 Material and methods

### 2.1 Inclusion criteria

**Figure 1:**
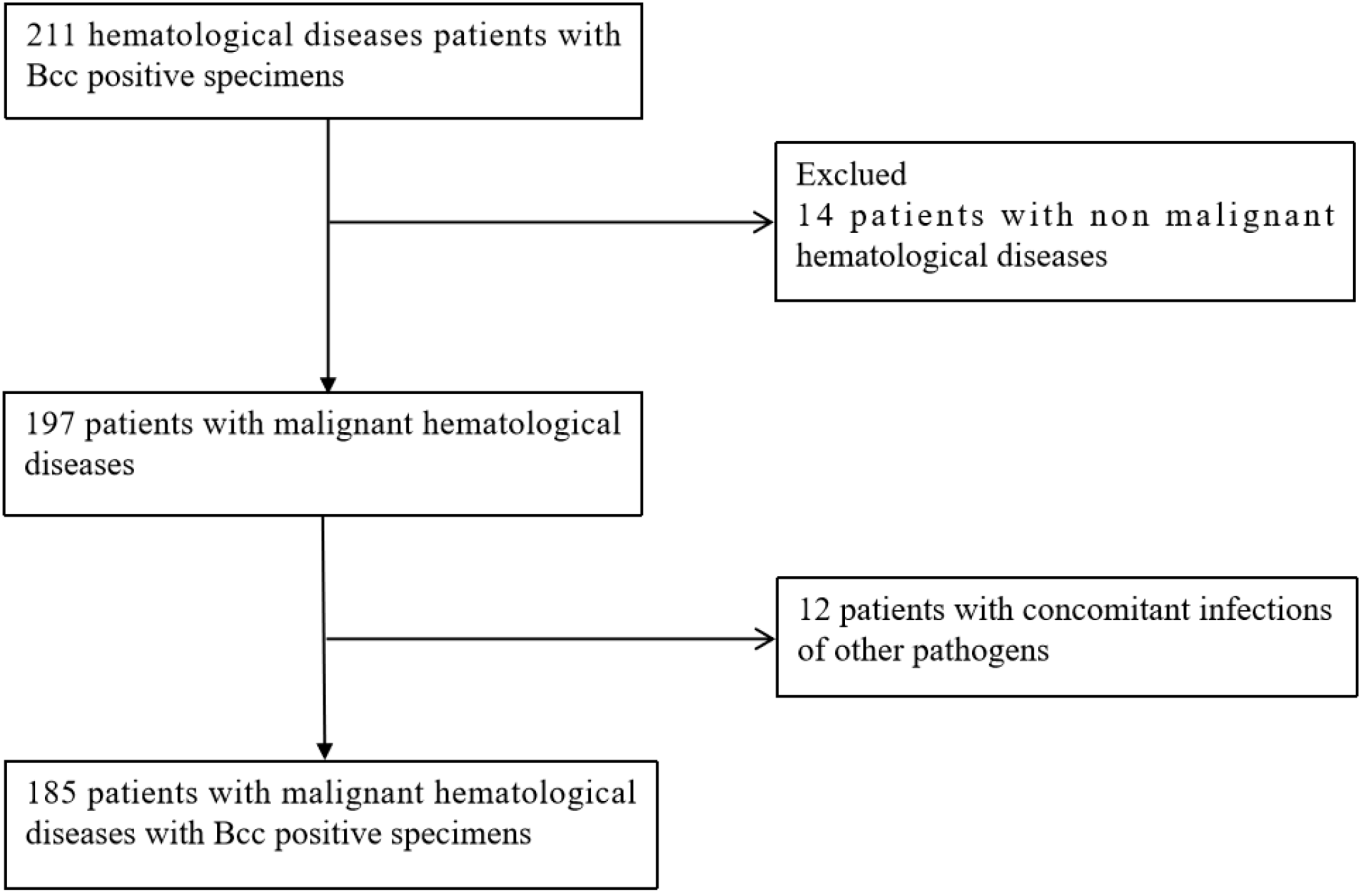
flow chart of patients included in the analysis.

A total of 211 hematologic patients with Bcc-positive cultures were initially identified at The First Affiliated Hospital of Soochow University between November 2019 and November 2022. After excluding 14 non-malignant cases and 12 cases co-infected with other pathogens (e.g., Fungal infection, Klebsiella pneumoniae, Staphylococcus aureus, Escherichia coli, Enterococcus faecium), 185 hematologic malignancy patients with Bcc colonization were included in the final analysis (**Figure 1**).

### 2.2 Data collection

#### 2.2.1 Clinical data

Gender, age; Prognosis; Neutrophil count, partial pressure of oxygen and carbon dioxide, liver function indicators, creatinine, etc.

#### 2.2.2 Definition of Bcc colonization

Patients in the Hematology Ward of the First Affiliated Hospital of Soochow University undergo throat swab screening every week. Therefore, according to the definitions of colonized bacteria by the Infectious Diseases Society of America (IDSA) and the European Society of Clinical Microbiology and Infectious Diseases (ESCMID) (Kalil et al., 2016; Woodhead et al., 2011), Bcc strains can be isolated from respiratory samples (such as sputum and bronchoalveolar lavage fluid), while meeting the following conditions:

1. Isolation of Bcc from respiratory samples (e.g., sputum, bronchoalveolar lavage fluid) without clinical signs of infection (e.g., fever, dyspnea, hemodynamic instability).
2. No significant elevation in inflammatory markers.
3. According to the IDSA/ATS guidelines for hospital acquired pneumonia, a single positive culture is not sufficient to diagnose infection. Therefore, patients need to have sputum culture or pharyngeal swab culture for two consecutive weeks to detect Bcc.

#### 2.2.3 Definition of post-colonization Bcc infection

1. Bcc was isolated from sterile sites (e.g. peripheral blood culture, Pleural effusion culture).
2. Non-sterile site cultures (e.g., sputum, throat swabs) were repeatedly positive with new/worsening respiratory symptoms, fever (>38°C), radiographic infiltrates, systemic inflammation (CRP > 50 mg/L or PCT > 0.5 ng/mL) (Kalil et al., 2016; Woodhead et al., 2011).

### 2.3 Cultivation and identification of Bcc species

All the clinical samples were inoculated on Blood Agar Plate (BAP), Chocolate Agar (CHOC) and incubated at 35°C for 48–72 hours.Species-level typing were performed using VITEK Mass Spectrometry, which can identify Bcc species.

### 2.4 Statistical analysis

Categorical variables were presented as numbers (percentages) and compared using the Chi-square test or Fisher’s exact test, as appropriate. Continuous variables were expressed as median with interquartile range (IQR) and compared using the Mann-Whitney U test. Univariate and multivariate logistic regression analyses were performed to identify factors associated with mortality, with results presented as odds ratios (OR) or hazard ratios (HR) with 95% confidence intervals (CI). A two-tailed *P*-value < 0.05 was considered statistically significant. All analyses were conducted using SPSS Statistics version 26.0 (IBM Corp., Armonk, NY, USA).

## 3 Results

### 3.1 Clinical characteristics of the study population

**Figure 2:**
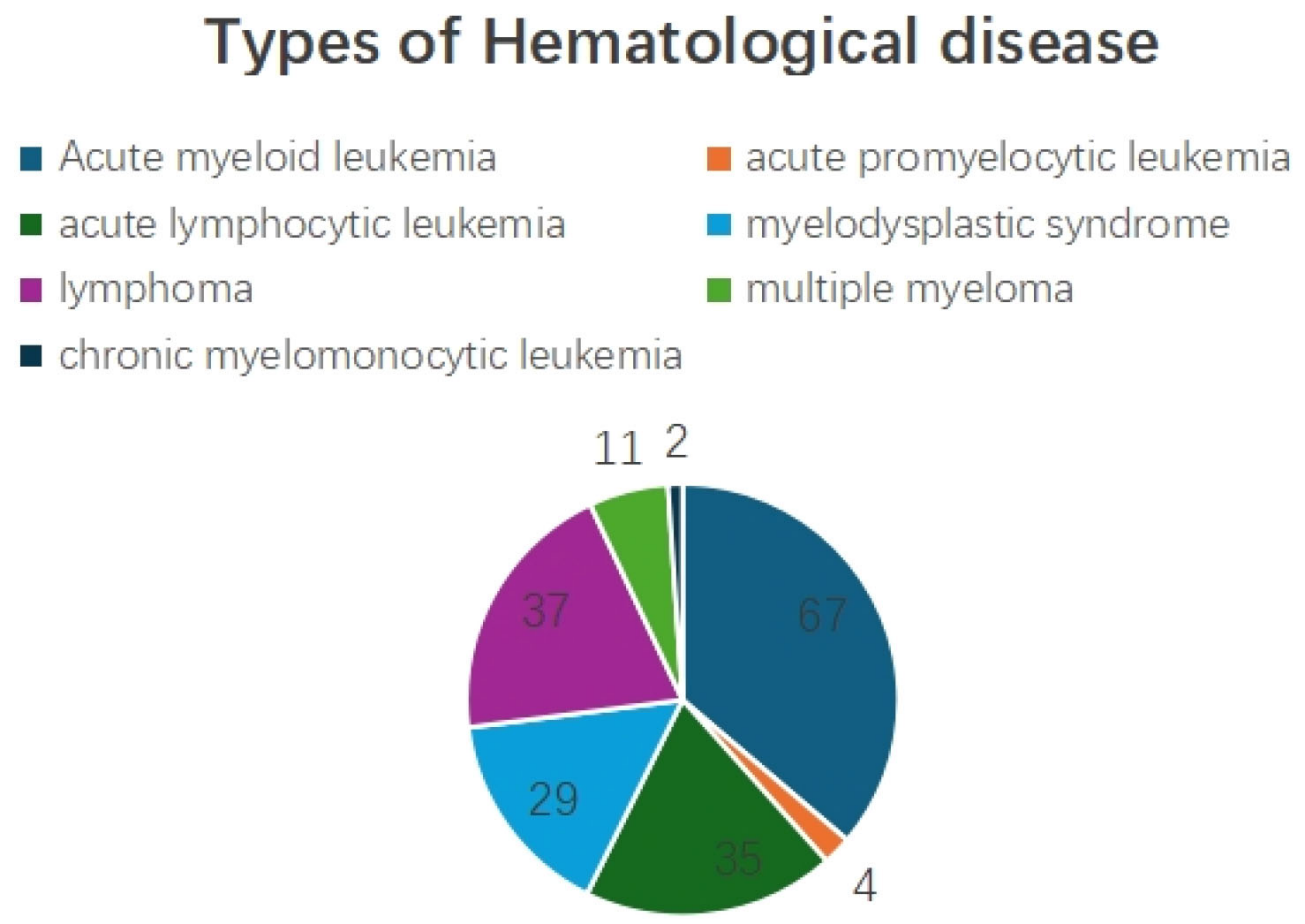
Types of Hematological disease.

Among the 185 patients, there were 72 cases of acute myeloid leukemia, 4 cases of acute promyelocytic leukemia, 32 cases of acute lymphocytic leukemia, 27 cases of myelodysplastic syndrome, 38 cases of lymphoma, 9 cases of multiple myeloma, and 3 cases of chronic myelomonocytic leukemia. (**Figure 2**).

A total of 185 patients with hematologic malignancies and confirmed Bcc colonization were included in the final analysis. Among them, 129 patients (69.7%) were classified as having Bcc colonization without signs of active infection, while 56 patients (30.3%) developed post-colonization Bcc infection. The cohort comprised 98 males (53%) and 87 females (47%), with a median age of 46 years (IQR: 30–55). The median length of hospital stay was 30 days (IQR:27-36). Among them, 139 patients(75.1%) were in immunosuppressive Status (e.g., calcineurin inhibitors, glucocorticoid, oral targeted drugs). Hematopoietic stem cell transplantation (HSCT) had been performed in 109 patients (58.9%), including 79 allogeneic and 30 autologous transplants. Neutropenia was present in 117 patients (63.2%), and 158 patients (85.4%) had indwelling catheters, all of which were peripherally inserted central catheter (PICC) catheters. Respiratory failure, hepatic insufficiency, and renal insufficiency were observed in 29 (15.7%), 68 (36.8%), and 35 (18.9%) patients, respectively. The 28-day mortality rate was 14.6% (27 patients). Detailed baseline characteristics are summarized in **Table 1**.

**Table 1:** Baseline data table of 185 patients with hematological malignancies whose BCC was cultured from specimens.

Characteristic	Total cohort (N=185)	Colonization only (N=129)	Post-colonization Bcc infection (N=56)
Demographics			
Age, years, median (IQR)	46(30, 55)	45 (29-54)	48 (32-57)
Male sex, n (%)	98 (53%)	61 (47.3%)	37 (6.6%)
Length of stay	30 (27,36)	28 (25,34)	31 (28,37)
Underlying Hematologic Malignancy, n (%)			
Acute Myeloid Leukemia	72 (38.9)	48 (37.2)	24 (42.9)
Acute promyelocytic leukemia	4 (2.2)	4 (3.1)	0 (0)
Acute Lymphocytic Leukemia	32 (17.3)	23 (17.8)	9 (16.1)
Myelodysplastic Syndrome	27 (14.6)	15 (11.6)	12 (21.4)
Lymphoma	38 (20.5)	25 (19.4)	13 (23.2)
multiple myeloma	9 (4.9)	5 (3.9)	4 (7.1)
chronic myelomonocytic leukemia	3 (1.6)	3 (2.3)	0 (0)
Clinical Status, n (%)			
Immunosuppressive Therapy	139 (75.1)	96 (74.4)	43 (76.8)
Hematopoietic Stem Cell Transplantation	109 (58.9)	77 (59.7)	33 (58.9)
- Allogeneic	100 (54.1)	76 (58.9)	24 (42.9)
- Autologous	9 (4.8)	0 (0)	9 (16.1)
Neutropenia status (<0.5×10^9^/L)	117 (63.2)	83 (64.3)	34 (60.7)
Indwelling Catheter (PICC)	158(85.4)	108 (83.7)	50 (89.3)
Organ Dysfunction, n (%)			
Respiratory failure	29 (15.7)	9 (7.0)	18 (32.1)
Liver dysfunction	68 (36.8)	38 (29.5)	30 (53.6)
Renal dysfunction	35 (18.9)	18 (14.0)	17 (30.4)
Outcome			
28-day Mortality, n (%)	27 (14.6)	13 (10.1)	14 (25.0)

### 3.2 Univariate analysis of prognostic factors

Univariate logistic regression analysis identified several factors significantly associated with poor prognosis in Bcc-colonized patients with hematologic malignancies. These included post-colonization Bcc infection (OR = 2.974, 95%CI: 1.293–6.844, *P* = 0.01), respiratory failure (OR = 20.683, 95%CI: 7.778–55.001, *P* < 0.001), hepatic insufficiency(OR = 8.266, 95%CI: 3.136–21.79, *P* < 0.001), and renal insufficiency (OR = 4.696, 95%CI: 1.951–11.303, *P* = 0.001). No significant associations were found for gender, age, immunosuppressive Status, HSCT status, or neutropenia. The complete univariate analysis is presented in **Table 2**.

**Table 2:** Univariate analysis of prognosis of patients with hematological malignancies combined with Bcc colonization.

Variable	Survival (N=158)	Died (N=27)	Odds ratio (OR)	95% CI for OR	*P*-value
Gender, Male	82	16	1.348	(0.589, 3.088)	0.48
Age ≥60 years	17	6	1.011	(0.984, 1.039)	0.424
Immunosuppressive Status	119	20	0.936	(0.4, 5.789)	0.89
Post-Colonization Bcc Infection	42	14	2.974	(1.293, 6.844)	0.01*
Hematopoietic stem cell transplantation status	95	14	1.4	(0.617, 3.177)	0.421
Neutropenia status	101	16	0.821	(0.357, 1.899)	0.643
Respiratory failure	12	17	20.683	(7.778, 55.001)	<0.001*
Liver dysfunction	47	21	8.266	(3.136, 21.79)	<0.001*
Renal dysfunction	23	12	4.696	(1.951, 11.303)	0.001*

### 3.3 Multivariate analysis of independent risk factors

In the multivariate Cox regression model, post-colonization Bcc infection remained an independent risk factor for mortality (HR = 2.855, 95%CI: 1.214–6.715,*P* = 0.016). Other variables, including gender, age, HSCT status, and neutropenia, did not show significant independent effects on prognosis. The results are detailed in **Table 3**.

**Table 3:** Multivariate analysis of prognosis of patients with hematological malignancies combined with Bcc colonization.

Variable	Hazard ratio (HR)	95% CI for HR	*P*-value
Gender, Male	1.151	(0.485, 2.731)	0.75
Age ≥60 years	1.004	(0.976, 1.034)	0.77
Post-Colonization Bcc Infection	2.855	(1.214, 6.715)	0.016*
Hematopoietic stem cell transplantation status	1.39	(0.594, 3.251)	0.448
Neutropenia status	0.874	(0.368, 2.076)	0.761

### 3.4 Subgroup analysis in hematopoietic stem cell transplant recipients

Among the 109 patients who had undergone HSCT, subgroup analysis revealed that post-colonization Bcc infection was a significant independent risk factor for mortality (HR = 3.733, 95% CI: 1.179–11.824, *P* = 0.025). Full results of the subgroup analysis are provided in **Table 4**.

**Table 4:** Subgroup analysis in hematopoietic stem cell transplant recipients.

Variable	Total (N=109)	Survival (N=95)	Died (N=14)	Hazard ratio (HR)	95% CI for HR	*P*-value
Gender, Male	59	51	8	2.48	(0.835, 7.361)	0.102
Post-Colonization Bcc Infection	33	25	8	3.733	(1.179, 11.824)	0.025*
Neutropenia status	72	63	9	0.426	(0.52. 4.708)	0.426
Respiratory failure	13	3	10	0.714	(0.153, 3.334)	0.669
Liver dysfunction	33	22	11	1.621	(0.575, 4.567)	0.361
Renal dysfunction	19	13	6	1.091	(0.27, 4.408)	0.903

## 4 Discussion

The Burkholderia cepacia complex represents a group of metabolically versatile, gram-negative bacteria that are widely distributed in the environment and recognized as opportunistic pathogens in immunocompromised hosts, including plant pathogens and human pathogens (Sawana et al., 2014). Among them, human pathogens can be divided into two categories. One is Burkholderia pseudomallei complex organisms (Bpc), which can cause glanders, including Burkholderia pseudomallei and Burkholderia pseudomallei; Another type is Burkholderia cepacia complex (Bcc), which belongs to environmental saprophytic bacteria and is also an important pathogen causing nosocomial infections (Sawana et al., 2014). If not controlled in a timely manner, Bcc infection can not only lead to severe pneumonia, but sometimes even progress to “onion syndrome”, which is characterized by fever, rapidly progressing necrotic pneumonia, and bacteremia, with a high mortality rate (Frost et al., 2019). In the past, research on infections caused by Bcc mainly focused on patients with cystic fibrosis (Lord et al., 2020), and in recent years, there have also been related studies on patients in intensive care units (Karakoc Parlayan et al., 2025). However, its role in patients with hematologic malignancies—a population characterized by profound immunosuppression—remains inadequately characterized (Jia et al., 2022).Our study demonstrates that progression from Burkholderia cepacia complex colonization to active infection is a pivotal clinical event, independently associated with increased mortality in patients with hematologic malignancies. This risk is profoundly amplified in the setting of hematopoietic stem cell transplantation. These findings underscore the critical need to distinguish between mere colonization and invasive disease in the management of this immunocompromised population.

Future research should also focus on the prognostic implications of Bcc species-level differentiation. The majority of bacterial strains in our study population were Burkholderia cepacia. Most patients had essentially used third-generation cephalosporin or even carbapenem antibiotics during their treatment. So, our study did not stratify outcomes by specific Bcc species, which may vary in virulence, and antibiotic resistance profiles. A study found that antibiotic sensitivity varies among different species and between cystic fibrosis and non-cystic fibrosis isolates. However, patients carrying B. cenocepacia, B. multivorans, and other Bcc species did not exhibit significant differences in lung function (Kenna et al., 2017). Another study on clinical isolates of Bcc also found that the distribution of Bcc isolates varies among different specimen types, and different Bcc isolates exhibit differences in their antibacterial susceptibility *in vitro* (Li et al., 2025). Therefore, the impact of different Bcc strains on the prognosis of patients with hematological malignancies remains unclear. Applying molecular typing techniques such as whole-genome sequencing to future research can clarify whether specific bacterial species independently lead to progression from colonization to infection, drug resistance, or death (Sawana et al., 2014).

The vulnerability of hematologic malignancy patients to Bcc is multifaceted. Patients with hematologic malignancies belong to immunocompromised populations who receive cytotoxic drugs, targeted therapies, and immunosuppressants during treatment, which can lead to neutropenia or even neutropenic conditions. Additionally, prophylactic antimicrobial therapy is required during the treatment period. This may be attributed to severe immunesuppression that is almost universally present in patients when treating hematological malignancies. The use of hormones, high-dose chemotherapy, pre-treatment regimens for HSCT, and disease targeted therapy can all lead to severe neutropenia and mucosal barrier damage - the main risk factors for opportunistic infections. Furthermore, patients with hematologic malignancies often require peripherally inserted central catheters (PICCs), ports, or other indwelling catheters during therapy (Abdallah et al., 2018), all of which increase the risk of Bcc colonization or infection. It seems that the high prevalence of indwelling devices provides a surface for Bcc biofilm formation, a key virulence trait that confers protection from both host defenses and antimicrobial agents (Liu et al., 2024; Magizhvannan and Veerappapillai, 2025).Therefore, studies have found that Bcc bacteremia in non-cystic fibrosis patients is mostly related to central venous catheter, and early removal of the catheter is crucial in treatment (Lee et al., 2020; Chang et al., 2022). In our center, almost everyone are PICCs, chosen specifically for this patient population due to their relative safety profile and ease of removal if infection is suspected, which is a critical management step. More importantly, PICC insertion and maintenance follow a strict, protocol-driven care bundle (including maximal sterile barrier at insertion, dedicated line care teams, and standardized dressing changes). This standardization, recommended by recent guidelines to minimize central line-associated bloodstream infections (CLABSIs) (Lebeaux et al., 2014), significantly reduces variability in nursing measures.

Our results corroborate and extend previous findings. The association between organ dysfunction (respiratory, hepatic, renal) and poor prognosis is consistent with studies of Bcc bacteremia in other settings (Lee et al., 2020).Previous studies have shown that advanced age, steroid use, immunosuppressive drugs, chemotherapy, higher antibiotic usage, type 2 diabetes, and invasive procedures are potential risk factors for bloodstream infections caused by Burkholderia cenocepacia (Chang et al., 2022; Siddiqui et al., 2022). A Korean retrospective study (1997–2016) found that most cases of Bcc bacteremia were nosocomial infections, with central venous catheter-related infections accounting for nearly half of the cases. Female gender, liver cirrhosis, septic shock, and catheter-related infections were independent risk factors for 30-day mortality; notably, liver cirrhosis significantly worsened prognosis in cases of Bcc bacteremia (Lee et al., 2020). This may be due to impaired antibiotic clearance rates and increased toxicity risks associated with liver metabolic dysfunction. In our study, hepatic insufficiency was associated with an eightfold increase in mortality risk (*P* < 0.001), reinforcing the importance of organ function in sepsis outcomes.

For HSCT recipients, Bcc post-colonization infection conferred a particularly grave prognosis. This is consistent with a 2022 study by Jia et al., which reported that Bcc-infected HSCT patients had significantly higher mortality, with septic shock and procalcitonin >10 µg/L identified as independent death predictors (Jia et al., 2022). Our subgroup analysis further validates that HSCT status amplifies the risk of Bcc-related mortality, highlighting a critical subpopulation for intensified surveillance.

This study indicates that when patients are found to be colonized with Bcc, healthcare workers need to assist in strengthening patients’ personal hygiene management, particularly oral hygiene. The study statistics reveal that 58.9% (33/56) of patients initially tested positive for Bcc in throat swabs, followed by detection in relatively cleaner specimens such as sputum, blood, bronchoalveolar lavage fluid, or wound secretions, marking a transition from Bcc colonization to infection. The study found that patients with Bcc colonization followed by infection have a poorer prognosis. However, different scholars have different opinions on targeted decolonization strategies for carriers of multidrug-resistant Gram negative bacteria (MDR-GNB), and their data is insufficient to provide strong recommendations for decolonization. (Tacconelli et al., 2019) Our findings highlight that progression from colonization to infection significantly increases mortality, particularly in HSCT recipients, raising the possibility that preemptive antimicrobial intervention might be beneficial in high-risk subgroups. Indiscriminate antibiotic use may promote further resistance without clear survival benefit. Instead, we advocate for a risk-stratified approach: for highest-risk patients (e.g., post-HSCT, prolonged neutropenia), targeted prophylaxis guided by local susceptibility patterns could be considered, while emphasizing non-pharmacological measures—enhanced surveillance, strict catheter care, and environmental control. (Kalil et al., 2016; Tacconelli et al., 2019).

To synthesize our findings into a clinically actionable framework, we have summarized the key risk factors, their underlying mechanisms, and proposed management strategies in **Table 5**. This synthesis emphasizes that prevention and early intervention are paramount.

**Table 5:** Risk factors and clinical management recommendations for post-colonization burkholderia cepacia complex infection in patients with hematologic malignancies.

Risk factor	Associated patient population	Clinical pecommendation
HSCT	Particularly allogeneic recipients; state of profound immunosuppression.	Implement enhanced periodic screening (e.g., weekly pharyngeal swabs); consider colonized patients as high-risk and intensify monitoring for early signs of infection.
Neutropenia	Patients undergoing intensive chemotherapy; risk correlates with duration.	Include anti-Bcc coverage in the empirical antibiotic regimen for febrile neutropenia; provide supportive care to shorten the duration of neutropenia.
Organ Insufficiency	Patients with hepatic, renal insufficiency, or respiratory failure.	Closely monitor organ function; adjust antibiotic dosing based on hepatic/renal function; obtain lower respiratory tract specimens for microbiological diagnosis early in patients with respiratory failure.
Indwelling Medical Devices	Patients with central venous catheters, urinary catheters, etc.	Maintain strict aseptic technique and device care; consider catheter-related infection in cases of unexplained fever and evaluate the need for catheter removal promptly.
Prior Respiratory Tract Colonization	Patients with positive screening results (e.g., pharyngeal swab, sputum culture).	Establish active surveillance screening protocols (e.g., weekly pharyngeal swabs); implement contact precautions for colonized patients and enhance environmental disinfection to prevent nosocomial transmission.

Based on our findings and existing evidence, we propose a pragmatic infection control bundle for high-risk hematology units: (1) Education of the staff for environmental cleaning and disinfection. Perform weekly hand hygiene audits. (2) Rigorous management of aqueous environments and medical devices(Such as ultrasound gel) (Angrup et al., 2022). Sterile covers were used for the ultrasound probes to prevent the gel touching the patient’s skin. (3) At least once a week surveillance with throat swabs in HSCT recipients and patients with profound neutropenia (Jia et al., 2022). (4)Enhanced source control including mupirocin nasal drop and chlorhexidine mouthwash care for colonized patients. (Ghaffary et al., 2024) (5)Pre-emptive contact isolation for colonized patients to limit potential transmission (Baker and Satlin, 2016). (6)Prompt diagnostic escalation upon symptom development, with expedited culture from sterile sites. Removal of central venous catheter in time (Lee et al., 2020).

Several limitations of our study warrant consideration. Our study is a single-center retrospective design with a limited sample size, especially in the subgroup undergoing hematopoietic stem cell transplantation, which may affect statistical power and limit the generalizability of the results. Multi-center, large-sample prospective studies are needed to further validate these findings and explore more accurate risk prediction models. Detailed antibiotic exposure and catheter parameters need be included. Furthermore, we will continue to study the relationship between the identification of Bcc species and patient prognosis in the future.

## 5 Conclusion

In conclusion, this study establishes Bcc post-colonization infection as a life-threatening complication in hematologic malignancy patients. Vigilant surveillance, strict infection control, and pre-emptive strategies are essential for high-risk individuals, particularly HSCT recipients. Future prospective, multicenter studies incorporating molecular typing and correlative microbiome analyses are needed to validate these findings and to develop more precise risk prediction models and targeted interventions.

## Data Availability

The original contributions presented in the study are included in the article/supplementary material. Further inquiries can be directed to the corresponding authors.
